# Supplementary material for: Epitope and HLA specificity of human TCRs against Plasmodium falciparum circumsporozoite protein
Source: J Exp Med. 2025 Jul 10;222(9):e20250044. doi: 10.1084/jem.20250044 (PMC12243953; doi:10.1084/jem.20250044)
Supplement: Table S1 — contains the TCR gene sequence information and reactivity data of all TCRs that have been screened in this study. [file jem_20250044_tables1.docx]

**Table S1. Sequence features, TCR expression, and reactivity of cloned and expressed TCRs from CD4^+^ and CD8^+^ T cells.**

| **TCR ID** | **Donor** | **Time point** | **Population** | **TRBV** | **TRBJ** | **TRBC** | **TRAV** | **TRAJ** | **TCR exp.** | **Reactivity** |
| --- | --- | --- | --- | --- | --- | --- | --- | --- | --- | --- |
| T21F7x1604 | F7 | II+28 | CD4 | 7-2 | 2-7 | 2 | 10-1 | 18 | 32.7 | Non-CSP-reactive |
| T21F9x2086 | F9 | II+28 | CD4 | 6-1 | 2-7 | 2 | 14DV4 | 13 | 63 | CS.T3 |
| T21F7x1777 | F7 | II+28 | CD4 | 7-2 | 2-1 | 2 | 10-1 | 18 | 56.4 | Non-CSP-reactive |
| T21F7x1643 | F7 | II+28 | CD4 | 7-2 | 2-7 | 2 | 10-1 | 18 | 66.8 | Non-CSP-reactive |
| T21F4x3646 | F4 | II+28 | CD4 | 20-1 | 2-5 | 2 | 17-1 | 50 | 41.5 | Non-CSP-reactive |
| T21F3x2835 | F3 | II+28 | CD4 | 12-3 | 1-5 | 1 | 17-1 | 11 | 71.6 | Th2R |
| T21F4x3818 | F4 | II+28 | CD4 | 19-1 | 2-7 | 2 | 13-1 | 47 | 27.8 | Non-CSP-reactive |
| T21F3x2797 | F3 | II+28 | CD4 | 5-1 | 2-6 | 2 | 12-1 | 20 | 42.8 | CSP61 |
| T21F3x2855 | F3 | II+28 | CD4 | 5-1 | 2-6 | 2 | 12-1 | 20 | 30.8 | CSP61 |
| T21F3x2742 | F3 | II+28 | CD4 | 5-1 | 2-6 | 2 | 12-1 | 20 | 69.5 | CSP61 |
| T21F3x2893 | F3 | II+28 | CD4 | 3-1 | 2-2 | 2 | 19-1 | 26 | 45.9 | CSP61 |
| T21F4x3685 | F4 | II+28 | CD4 | 20-1 | 2-2 | 2 | 9-2 | 49 | 78.9 | CS.T3 |
| T21F4x3722 | F4 | II+28 | CD4 | 27-1 | 1-4 | 1 | 1-2 | 33 | 69.7 | CSP81 |
| T21F7x1698 | F7 | II+28 | CD4 | 30-1 | 2-2 | 2 | 29DV5 | 18 | 64.3 | T* |
| T21F4x3701 | F4 | II+28 | CD4 | 20-1 | 2-5 | 2 | 9-2 | 57 | 68.4 | CS.T3 |
| T21F3x3023 | F3 | II+28 | CD4 | 14-1 | 1-2 | 1 | 19-1 | 43 | 46.9 | Non-CSP-reactive |
| T21F3x2714 | F3 | II+28 | CD4 | 30-1 | 1-5 | 1 | 24-1 | 28 | 75.3 | T1 |
| T21F3x2705 | F3 | II+28 | CD4 | 30-1 | 1-3 | 1 | 24-1 | 32 | 68.9 | T1 |
| T21F3x2851 | F3 | II+28 | CD4 | 30-1 | 1-6 | 1 | 3-1 | 36 | 81 | T1 |
| T21F3x2777 | F3 | II+28 | CD4 | 30-1 | 1-2 | 1 | 36DV7 | 54 | 66.1 | T1 |
| T21F3x2838 | F3 | II+28 | CD4 | 12-3 | 1-5 | 1 | 17-1 | 11 | 66.7 | Th2R |
| T21F3x2968 | F3 | II+28 | CD4 | 12-4 | 1-2 | 1 | 17-1 | 33 | 28.8 | Non-CSP-reactive |
| T21F3x2862 | F3 | II+28 | CD4 | 6-4 | 1-2 | 1 | 17-1 | 11 | 67.6 | Th2R |
| T21F4x3702 | F4 | II+28 | CD4 | 20-1 | 2-7 | 2 | 9-2 | 58 | 65.4 | CS.T3 |
| T21F3x2939 | F3 | II+28 | CD4 | 12-3 | 1-3 | 1 | 26-1 | 48 | 76.1 | Non-CSP-reactive |
| T21F3x3026 | F3 | II+28 | CD4 | 7-2 | 2-3 | 2 | 26-1 | 23 | 53.3 | Non-CSP-reactive |
| T21F4x3706 | F4 | II+28 | CD4 | 20-1 | 2-1 | 2 | 9-2 | 10 | 86 | CS.T3 |
| T21F3x2949 | F3 | II+28 | CD4 | 12-3 | 1-2 | 1 | 23DV6 | 23 | 43.3 | Non-CSP-reactive |
| T21F3x2863 | F3 | II+28 | CD4 | 7-2 | 1-5 | 1 | 17-1 | 11 | 56 | Th2R |
| T21F3x2903 | F3 | II+28 | CD4 | 7-2 | 2-1 | 2 | 17-1 | 11 | 49.5 | Th2R |
| T21F3x2996 | F3 | II+28 | CD4 | 5-1 | 1-5 | 1 | 29DV5 | 40 | 71.8 | Non-CSP-reactive |
| T21F3x2911 | F3 | II+28 | CD4 | 18-1 | 1-2 | 1 | 13-1 | 53 | 72.2 | Th2R |
| T21F3x2865 | F3 | II+28 | CD4 | 7-2 | 2-3 | 2 | 35-1 | 17 | 38.8 | Non-CSP-reactive |
| T21F3x3008 | F3 | II+28 | CD4 | 27-1 | 1-6 | 1 | 26-2 | 40 | 60.1 | Non-CSP-reactive |
| T21F4x3724 | F4 | II+28 | CD4 | 20-1 | 2-2 | 2 | 35-1 | 49 | 56.4 | Non-CSP-reactive |
| T21F7x1635 | F7 | II+28 | CD4 | 5-8 | 1-1 | 1 | 29DV5 | 45 | 58.5 | Non-CSP-reactive |
| T21F3x2915 | F3 | II+28 | CD4 | 7-2 | 1-5 | 1 | 17-1 | 11 | 41.5 | Th2R |
| T21F9x2073 | F9 | II+28 | CD4 | 6-1 | 1-5 | 1 | 29DV5 | 53 | 64 | Non-CSP-reactive |
| T21F9x2018 | F9 | II+28 | CD4 | 12-4 | 2-7 | 2 | 39-1 | 48 | 61.1 | Non-CSP-reactive |
| T21F4x3758 | F4 | II+28 | CD4 | 28-1 | 2-1 | 2 | 41-1 | 57 | 24 | Non-CSP-reactive |
| T21F4x3762 | F4 | II+28 | CD4 | 20-1 | 2-5 | 2 | 9-2 | 35 | 68.4 | CS.T3 |
| T21F4x3653 | F4 | II+28 | CD4 | 28-1 | 2-1 | 2 | 35-1 | 28 | 60.3 | Non-CSP-reactive |
| T21F3x2917 | F3 | II+28 | CD4 | 7-7 | 1-1 | 1 | 8-4 | 15 | 34.6 | Th2R |
| T21F3x2907 | F3 | II+28 | CD4 | 5-1 | 2-1 | 2 | 38-2-DV8 | 52 | 75 | Non-CSP-reactive |
| T21F3x2931 | F3 | II+28 | CD4 | 3-1 | 1-3 | 1 | 17-1 | 34 | 59.2 | Th2R |
| T21F3x2795 | F3 | II+28 | CD4 | 3-1 | 2-4 | 2 | 8-3 | 45 | 12 | Non-CSP-reactive |
| T21F3x2945 | F3 | II+28 | CD4 | 12-3 | 1-5 | 1 | 17-1 | 11 | 63.9 | Th2R |
| T21F3x3020 | F3 | II+28 | CD4 | 12-3 | 1-1 | 1 | 8-4 | 50 | 76 | Non-CSP-reactive |
| T21F4x3712 | F4 | II+28 | CD4 | 5-4 | 2-7 | 2 | 8-4 | 49 | 63.2 | Non-CSP-reactive |
| T21F3x3018 | F3 | II+28 | CD4 | 14-1 | 1-2 | 1 | 9-2 | 20 | 70.3 | Non-CSP-reactive |
| T21F3x2960 | F3 | II+28 | CD4 | 12-3 | 1-5 | 1 | 17-1 | 11 | 71.5 | Th2R |
| T21F4x3791 | F4 | II+28 | CD4 | 20-1 | 2-1 | 2 | 9-2 | 32 | 79.8 | Non-CSP-reactive |
| T21F3x2975 | F3 | II+28 | CD4 | 5-1 | 2-5 | 2 | 9-2 | 52 | 71.6 | Th2R |
| T21F4x3795 | F4 | II+28 | CD4 | 20-1 | 1-4 | 1 | 13-1 | 29 | 76.2 | EBV |
| T21F3x3019 | F3 | II+28 | CD4 | 5-1 | 2-5 | 2 | 9-2 | 52 | 69 | Th2R |
| T21F4x3804 | F4 | II+28 | CD4 | 20-1 | 2-1 | 2 | 12-3 | 45 | 68 | Non-CSP-reactive |
| T21F4x3682 | F4 | II+28 | CD4 | 20-1 | 2-4 | 2 | 13-1 | 53 | 61.8 | Th2R |
| T21F3x3049 | F3 | II+28 | CD4 | 30-1 | 1-1 | 1 | 13-2 | 31 | 70.4 | Non-CSP-reactive |
| T21F3x2731 | F3 | II+28 | CD4 | 30-1 | 1-5 | 1 | 23DV6 | 36 | 69.3 | T1 |
| T21F3x2717 | F3 | II+28 | CD4 | 30-1 | 2-7 | 2 | 36DV7 | 54 | 77.1 | T1 |
| T21F3x2989 | F3 | II+28 | CD4 | 5-1 | 2-5 | 2 | 9-2 | 52 | 74.9 | Th2R |
| T21F3x2898 | F3 | II+28 | CD4 | 5-1 | 2-6 | 2 | 9-2 | 49 | 82.5 | Non-CSP-reactive |
| T21F3x2807 | F3 | II+28 | CD4 | 30-1 | 2-3 | 2 | 26-1 | 27 | 74.2 | Non-CSP-reactive |
| T21F4x3806 | F4 | II+28 | CD4 | 20-1 | 2-2 | 2 | 8-3 | 15 | 31.4 | Non-CSP-reactive |
| T21F4x3825 | F4 | II+28 | CD4 | 20-1 | 2-5 | 2 | 9-2 | 10 | 68.9 | CS.T3 |
| T21F3x2801 | F3 | II+28 | CD4 | 20-1 | 2-7 | 2 | 13-1 | 15 | 61.7 | T* |
| T19F1x1257 | F1 | II+28 | T_EM_+T_EMRA_ | 27 | 2-1 | 2 | 2 | 30 | 16 | Non-CSP-reactive |
| T19F1x2356 | F1 | III+14 | T_EM_+T_EMRA_ | 12-3 | 1-1 | 1 | 29/DV5 | 43 | 58 | Non-CSP-reactive |
| T19F1x2391 | F1 | III+14 | T_EM_+T_EMRA_ | 11-2 | 2-1 | 2 | 13-2 | 47 | 24 | Non-CSP-reactive |
| T19F2x1302 | F2 | II+28 | T_EM_+T_EMRA_ | 27 | 2-7 | 2 | 12-3 | 13 | 71 | EBV reactive |
| T19F3x874 | F3 | III+14 | T_EM_+T_EMRA_ | 7-9 | 1-6 | 1 | 41 | 57 | 41 | Non-CSP-reactive |
| T19F3x906 | F3 | III+14 | T_EM_+T_EMRA_ | 27 | 2-5 | 2 | 29/DV5 | 47 | 45 | Non-CSP-reactive |
| T19F4x3235 | F4 | III+14 | T_EM_+T_EMRA_ | 4-3 | 2-2 | 2 | 12-2 | 49 | 29 | Non-CSP-reactive |
| T19F4x3308 | F4 | III+14 | T_EM_+T_EMRA_ | 12-3 | 1-2 | 1 | 12-2 | 49 | 69 | Non-CSP-reactive |
| T19F4x3320 | F4 | III+14 | T_EM_+T_EMRA_ | 12-3 | 1-5 | 1 | 12-3 | 49 | 47 | Non-CSP-reactive |
| T19F5x1657 | F5 | III+14 | T_EM_+T_EMRA_ | 18 | 1-1 | 1 | 41 | 48 | 61 | Non-CSP-reactive |
| T19F5x1793 | F5 | III+14 | Tet+ | 28 | 1-6 | 1 | 26-2 | 48 | 83 | Non-CSP-reactive |
| T19F5x1850 | F5 | III+14 | Tet+ | 28 | 2-3 | 2 | 12-2 | 45 | 59 | Non-CSP-reactive |
| T19F5x2130 | F5 | III+14 | T_EM_+T_EMRA_ | 28-1 | 2-1 | 2 | 12-1 | 49 | 42 | Non-CSP-reactive |
| T19F5x2143 | F5 | III+14 | T_EM_+T_EMRA_ | 7-6 | 1-5 | 1 | 26-1 | 49 | 60 | Non-CSP-reactive |
| T19F5x2201 | F5 | III+14 | Tet+ | 9-1 | 2-1 | 2 | 13-1 | 15 | 63 | Non-CSP-reactive |
| T19F5x2242 | F5 | III+14 | Tet+ | 7-9 | 1-6 | 1 | 8-2 | 12 | 25 | Non-CSP-reactive |
| T19F5x2245 | F5 | III+14 | Tet+ | 28-1 | 2-3 | 2 | 12-1 | 49 | 63 | Non-CSP-reactive |
| T19F5x2251 | F5 | III+14 | Tet+ | 27 | 1-2 | 1 | 19 | 44 | 52 | Non-CSP-reactive |
| T19F5x4251 | F5 | II+28 | T_EM_+T_EMRA_ | 20-1 | 2-7 | 2 | 27 | 24 | 50 | Non-CSP-reactive |
| T19F5x4261 | F5 | II+28 | T_EM_+T_EMRA_ | 28 | 2-3 | 2 | 12-2 | 49 | 54 | Non-CSP-reactive |
| T19F5x4313 | F5 | II+28 | T_EM_+T_EMRA_ | 20-1 | 2-5 | 2 | 30 | 12 | 22 | Non-CSP-reactive |
| T19F5x4355 | F5 | II+28 | T_EM_+T_EMRA_ | 25-1 | 1-3 | 1 | 13-2 | 16 | 34 | Non-CSP-reactive |
| T19F5x4356 | F5 | II+28 | T_EM_+T_EMRA_ | 28 | 1-6 | 1 | 12-2 | 28 | 67 | Non-CSP-reactive |
| T19F5x4361 | F5 | II+28 | T_EM_+T_EMRA_ | 12-4 | 2-3 | 2 | 12-2 | 49 | 64 | Non-CSP-reactive |
| T19F5x4395 | F5 | II+28 | T_EM_+T_EMRA_ | 5-8 | 2-7 | 2 | 14/DV4 | 8 | 69 | Non-CSP-reactive |
| T19F5x4452 | F5 | II+28 | Tet+ | 5-6 | 2-3 | 2 | 13-2 | 48 | 48 | Non-CSP-reactive |
| T19F5x4469 | F5 | II+28 | Tet+ | 27 | 1-4 | 1 | 21 | 26 | 69 | Non-CSP-reactive |
| T19F5x4473 | F5 | II+28 | Tet+ | 7-6 | 1-4 | 1 | 26-2 | 43 | 66 | Non-CSP-reactive |
| T19F5x4501 | F5 | II+28 | Tet+ | 9 | 1-5 | 1 | 19 | 30 | 41 | Non-CSP-reactive |
| T19F5x4523 | F5 | II+28 | Tet+ | 12-4 | 2-7 | 2 | 1-1 | 41 | 25 | Non-CSP-reactive |
| T19F5x4529 | F5 | II+28 | Tet+ | 27 | 2-1 | 2 | 21 | 26 | 63 | Non-CSP-reactive |
| T19F5x4850 | F5 | II+28 | Tet+ | 19-1 | 2-1 | 2 | 12-2 | 42 | 28 | Non-CSP-reactive |
| T19F5x4886 | F5 | II+28 | Tet+ | 7-8 | 2-4 | 2 | 21-1 | 49 | 47 | Non-CSP-reactive |
| T19F5x4909 | F5 | II+28 | Tet+ | 20-1 | 2-7 | 2 | 8-6 | 52 | 62 | Non-CSP-reactive |
| T19F5x4912 | F5 | II+28 | Tet+ | 19-1 | 1-5 | 1 | 12-2 | 30 | 57 | Non-CSP-reactive |
| T20F2x2439 | F2 | III+14 | T_EM_+T_EMRA_ | 5-8 | 2-5 | 2 | 13-2 | 45 | 52 | Non-CSP-reactive |
| T20F2x2549 | F2 | III+14 | Tet+ | 10-2 | 2-7 | 2 | 12-1 | 37 | 53 | Non-CSP-reactive |
| T20F3x2177 | F3 | II+28 | T_EM_+T_EMRA_ | 2-1 | 2-7 | 2 | 13-1 | 24 | 62 | Non-CSP-reactive |
| T20F4x2630 | F4 | III+14 | T_EM_+T_EMRA_ | 3-1 | 1-5 | 1 | 12-2 | 23 | 51 | Non-CSP-reactive |
| T20F4x2658 | F4 | III+14 | T_EM_+T_EMRA_ | 12-3 | 2-7 | 2 | 26-1 | 20 | 68 | Non-CSP-reactive |
| T20F4x3312 | F4 | II+28 | CD8 | 7-9 | 1-2 | 1 | 29/DV5 | 52 | 30 | EBV reactive |
| T20F5x2724 | F5 | II+28 | Tet+ | 18-1 | 2-5 | 2 | 16-1 | 39 | 31 | Non-CSP-reactive |
| T20F5x2765 | F5 | II+28 | Tet+ | 6-2 | 2-7 | 2 | 10-1 | 34 | 35 | Non-CSP-reactive |
| T20F5x2820 | F5 | II+28 | Tet+ | 7-2 | 2-2 | 2 | 1-2 | 31 | 60 | Non-CSP-reactive |
| T20F5x2874 | F5 | II+28 | Tet+ | 5-6 | 2-3 | 2 | 4-1 | 5 | 67 | Non-CSP-reactive |
| T20F5x2913 | F5 | II+28 | T_EM_+T_EMRA_ | 11-3 | 2-1 | 2 | 12-2 | 20 | 36 | Non-CSP-reactive |
| T20F5x2984 | F5 | II+28 | T_EM_+T_EMRA_ | 3-1 | 2-1 | 2 | 8-4 | 54 | 55 | Non-CSP-reactive |
| T20F5x3019 | F5 | II+28 | T_EM_+T_EMRA_ | 20-1 | 2-5 | 2 | 8-2 | 12 | 56 | Non-CSP-reactive |
| T20F5x3039 | F5 | II+28 | T_EM_+T_EMRA_ | 15-1 | 2-1 | 2 | 22-1 | 35 | 57 | Non-CSP-reactive |
| T20F5x3873 | F5 | III+14 | T_EM_+T_EMRA_ | 10-3 | 2-5 | 2 | 8-3 | 13 | 53 | Non-CSP-reactive |
| T20F5x3992 | F5 | III+14 | T_EM_+T_EMRA_ | 6-5 | 1-2 | 1 | 4-1 | 41 | 73 | Non-CSP-reactive |
| T20F5x4028 | F5 | III+14 | T_EM_+T_EMRA_ | 7-9 | 2-1 | 2 | 19-1 | 12 | 20 | Non-CSP-reactive |
| T20F5x4029 | F5 | III+14 | T_EM_+T_EMRA_ | 19-1 | 2-7 | 2 | 19-1 | 42 | 36 | Non-CSP-reactive |
| T20F5x4035 | F5 | III+14 | T_EM_+T_EMRA_ | 6-2 | 1-5 | 1 | 41-1 | 45 | 54 | Non-CSP-reactive |
| T20F5x4038 | F5 | III+14 | T_EM_+T_EMRA_ | 3-1 | 1-1 | 1 | 38-2-DV8 | 40 | 63 | Non-CSP-reactive |
| T20F5x4097 | F5 | III+14 | Tet+ | 19-1 | 2-1 | 2 | 8-2 | 30 | 48 | Non-CSP-reactive |
| T20F5x4140 | F5 | III+14 | Tet+ | 18-1 | 2-7 | 2 | 12-1 | 44 | 37 | Non-CSP-reactive |
| T20F5x4162 | F5 | III+14 | Tet+ | 20-1 | 1-5 | 1 | 12-2 | 24 | 79 | Non-CSP-reactive |
| T20F5x4208 | F5 | III+14 | Tet+ | 6-2 | 1-1 | 1 | 12-2 | 12 | 55 | Non-CSP-reactive |
| T20F5x4215 | F5 | III+14 | Tet+ | 19-1 | 1-2 | 1 | 38-2-DV8 | 27 | 54 | Non-CSP-reactive |
| T19F4x2853 | F4 | III+14 | T_EM_+T_EMRA_ | 12-3 | 1-2 | 1 | 12-1 | 31 | 70.8 | Non-CSP-reactive |
| T19F4x2853 | F4 | III+14 | T_EM_+T_EMRA_ | 12-3 | 1-2 | 1 | 12-1 | 31 | 44.3 | Non-CSP-reactive |
| T19F5x4436 | F5 | II+28 | T_EM_+T_EMRA_ | 28-1 | 2-1 | 2 | 1-2 | 27 | 13.7 | Non-CSP-reactive |
| T19F4x3016 | F4 | III+14 | T_EM_+T_EMRA_ | 12-3 | 1-1 | 1 | 12-2 | 31 | 23.5 | Non-CSP-reactive |
| T19F3x915 | F3 | III+14 | Tet+ | 20-1 | 2-7 | 2 | 24-1 | 34 | 50.9 | Non-CSP-reactive |
| T19F5x4265 | F5 | II+28 | T_EM_+T_EMRA_ | 27-1 | 2-2 | 2 | 27-1 | 50 | 36.6 | Non-CSP-reactive |
| T19F3x856 | F3 | III+14 | T_EM_+T_EMRA_ | 12-4 | 1-2 | 1 | 35-1 | 50 | 38.5 | Non-CSP-reactive |
| T21F1x615 | F1 | II+28 | CD8 | 15-1 | 1-1 | 1 | 12-2 | 30 | 24.3 | Non-CSP-reactive |
| T21F3x649 | F3 | II+28 | CD8 | 12-4 | 1-1 | 1 | 29DV5 | 30 | 14.1 | Non-CSP-reactive |
| T21F3x661 | F3 | II+28 | CD8 | 5-1 | 2-7 | 2 | 25-1 | 32 | 56.2 | Non-CSP-reactive |
| T21F3x857 | F3 | II+28 | CD8 | 3-1 | 1-3 | 1 | 8-6 | 23 | 61.3 | Non-CSP-reactive |
| T21F3x863 | F3 | II+28 | CD8 | 20-1 | 2-7 | 2 | 24-1 | 34 | 78.6 | Non-CSP-reactive |
| T21F3x883 | F3 | II+28 | CD8 | 12-4 | 1-2 | 1 | 35-1 | 50 | 52.4 | Non-CSP-reactive |
| T21F3x1001 | F3 | II+28 | CD8 | 12-4 | 2-7 | 2 | 27-1 | 40 | 70.5 | Non-CSP-reactive |
| T21F3x1010 | F3 | II+28 | CD8 | 27-1 | 2-1 | 2 | 6-1 | 29 | 17.2 | Non-CSP-reactive |
| T21F3x1025 | F3 | II+28 | CD8 | 29-1 | 2-7 | 2 | 35-1 | 20 | 63 | Non-CSP-reactive |
| T21F3x1031 | F3 | II+28 | CD8 | 5-1 | 1-2 | 1 | 29DV5 | 40 | 29.5 | Non-CSP-reactive |
| T21F3x1037 | F3 | II+28 | CD8 | 12-4 | 2-4 | 2 | 22-1 | 29 | 55 | Non-CSP-reactive |
| T21F3x1053 | F3 | II+28 | CD8 | 5-5 | 1-3 | 1 | 22-1 | 48 | 77.6 | Non-CSP-reactive |
| T21F3x1101 | F3 | II+28 | CD8 | 5-1 | 1-2 | 1 | 17-1 | 53 | 61.4 | Non-CSP-reactive |
| T21F3x1113 | F3 | II+28 | CD8 | 5-1 | 1-1 | 1 | 19-1 | 57 | 59.2 | Non-CSP-reactive |
| T21F7x1757 | F7 | II+28 | CD8 | 30-1 | 2-2 | 2 | 12-2 | 15 | 57.3 | Non-CSP-reactive |
| T21F7x1811 | F7 | II+28 | CD8 | 19-1 | 2-1 | 2 | 12-1 | 8 | 30.9 | Non-CSP-reactive |
| T21F9x1889 | F9 | II+28 | CD8 | 7-2 | 1-2 | 1 | 36DV7 | 53 | 47 | CS.T3 |
| T21F7x1907 | F7 | II+28 | CD8 | 30-1 | 2-2 | 2 | 12-2 | 15 | 62.9 | Non-CSP-reactive |
| T21F9x2160 | F9 | II+28 | CD8 | 7-2 | 1-4 | 1 | 1-2 | 20 | 66.6 | Non-CSP-reactive |
| T21F7x2213 | F7 | II+28 | CD8 | 7-9 | 2-1 | 2 | 12-2 | 24 | 34.2 | Non-CSP-reactive |
| T21F7x2225 | F7 | II+28 | CD8 | 5-8 | 2-3 | 2 | 29DV5 | 45 | 51.2 | Non-CSP-reactive |
| T21F4x2344 | F4 | II+28 | CD8 | 5-1 | 2-7 | 2 | 1-2 | 33 | 69.4 | Non-CSP-reactive |
| T21F4x2345 | F4 | II+28 | CD8 | 20-1 | 2-1 | 2 | 26-1 | 9 | 64.6 | Non-CSP-reactive |
| T21F4x2376 | F4 | II+28 | CD8 | 4-1 | 1-2 | 1 | 8-3 | 35 | 13.1 | Non-CSP-reactive |
| T21F4x2396 | F4 | II+28 | CD8 | 19-1 | 2-7 | 2 | 22-1 | 24 | 58.3 | Non-CSP-reactive |
| T21F4x2402 | F4 | II+28 | CD8 | 20-1 | 2-2 | 2 | 39-1 | 16 | 49.9 | Non-CSP-reactive |
| T21F4x2507 | F4 | II+28 | CD8 | 12-3 | 2-2 | 2 | 1-2 | 33 | 68.7 | Non-CSP-reactive |
| T21F4x2512 | F4 | II+28 | CD8 | 20-1 | 2-7 | 2 | 13-1 | 13 | 46.8 | Non-CSP-reactive |
| T21F4x2528 | F4 | II+28 | CD8 | 5-1 | 2-1 | 2 | 17-1 | 57 | 51.7 | Non-CSP-reactive |
| T21F4x2494 | F4 | II+28 | CD8 | 27-1 | 1-2 | 1 | 1-2 | 6 | 61 | Non-CSP-reactive |
